# Preventing Disease

**Published:** 1880-09

**Authors:** 


**PREVENTING DISEASE.** — To inaugurate a healthy, moral condition of the community, we must prevent crime by educating the masses to its avoidance. This is the part of the true philanthropist; so is it the highest aim of the good physician, not to cure disease, but to prevent it. Such was the announcement in the very first line of the first number of our JOURNAL. On the title-page we intimated that the good time was coming, and we are gratified at some signs of its approach, infinitely more tangible than any Dr. Cummings can show that the world is near its end. In the great city of London, which now covers a space of one hundred and twenty-one square miles, on which live two and three quarter millions of people, one person out of every dozen dies in a workhouse, one out of every half-dozen in a charitable institution, one person out of every six is too poor to die in his own bed, and must rest in a pauper's grave. To what extent sickness and slow disease incapacitate the striving multitudes from supporting themselves, none can estimate better than the physician and the philanthropist. In view of so sad a result in the greatest city of the greatest nation on earth, the Registrar-General first looks for relief in improving the physical health of the teeming millions, and to this end aims at securing three things:

1. Pure air to breathe.
2. Pure water to drink.
3. A healthy soil to live upon.

